# The impact of population influx on infectious diseases – from the mediating effect of polluted air transmission

**DOI:** 10.3389/fpubh.2024.1344306

**Published:** 2024-07-30

**Authors:** Haifeng Fu, Chaoping Zhu

**Affiliations:** ^1^School of Accounting and Finance, Jiangxi College of Foreign Studies, Nanchang, Jiangxi, China; ^2^School of Transportation Management, Jiangxi Vocational and Technical College of Communications, Nanchang, Jiangxi, China; ^3^School of Software, Jiangxi Normal University, Nanchang, Jiangxi, China

**Keywords:** population influx, air pollution, infectious diseases, personal income, medical resources, educational resources

## Abstract

The global population influx during the COVID-19 pandemic poses significant challenges to public health, making the prevention and control of infectious diseases a pressing concern. This paper aims to examine the impact of population influx on the spread of infectious diseases, with a specific emphasis on the mediating role of air pollution in this process. A theoretical analysis is conducted to explore the relationship between population influx, air pollution, and infectious diseases. Additionally, we establish a series of econometric models and employ various empirical tests and analytical techniques, including mediation effect test, threshold effect test, and systematic GMM test, to evaluate our hypotheses. The results indicate that: (1) Population influx directly and indirectly impacts infectious diseases. Specifically, population influx not only directly elevates the risk of infectious diseases, but also indirectly increases the incidence rate of infectious diseases by intensifying air pollution. (2) The impact of population inflow on infectious diseases exhibits regional heterogeneity. Compared to central and western China, the eastern regions exhibit a significantly higher risk of infectious diseases, exceeding the national average. (3) External factors influence the relationship between population influx and infectious diseases differently. Personal income and medical resources both help mitigate the risk of infectious diseases due to population influx, with medical resources having a more substantial effect. Contrary to expectations, abundant educational resources have not reduced the risk, instead, they have exacerbated the risk associated with population influx. This paper provides a scientific basis for formulating effective strategies for the prevention and control of infectious diseases.

## 1 Introduction

In the 21st century, globalization and urbanization have accelerated population influx. According to the Seventh National Census in 2020, the migrant population in China reached 376 million, an increase of 69.73% compared to 2010, accounting for approximately 26.69% of the total population. The intra-provincial migrant population in China is 251 million, an increase of 115 million compared to 2010, while the inter-provincial migrant population is 125 million, an increase of 39 million compared to 2010. This significant population influx has profoundly impacted public health security, particularly in the prevention and control of infectious diseases. In 2020, 2.67 million cases of Class A and B infectious diseases were reported nationwide, with 26,000 deaths. The top five reported morbidity cases were viral hepatitis, tuberculosis, syphilis, gonorrhea, and COVID-19, accounting for 92.2% of the total morbidity. The top five reported causes of death were AIDS, COVID-19, tuberculosis, viral hepatitis, and rabies, which account for 99.5% of total deaths. The spread of these diseases is closely related to population influx and a major threat to global health.

Airborne infectious diseases are transmitted through airborne particles such as particulate matter and aerosols, which may contain viruses, bacteria, or other microorganisms that enter the body through the respiratory tract. Examples of such diseases include COVID-19, tuberculosis, influenza, and measles. The population influx exacerbates the risk of diseases transmission, thus creating favorable conditions for the spread of airborne infectious diseases. In 2020, 135 prefecture-level cities in China, accounting for 40.1%, failed to meet air quality standards. Particulate matter in polluted air can provide an environment for pathogens to survive and reproduce, which indirectly promotes the spread of infectious diseases. This study explores the relationship between population mobility, air pollution and infectious diseases, thereby offering insights that contribute to the development of more effective public health strategies and interventions. The findings provide a scientific basis for optimizing resource allocation and prioritizing the enhancement of healthcare resources to improve the public health system’s capacity to respond to infectious disease outbreaks.

The relationship among population influx, air pollution, and infectious diseases, has been extensively studied, focusing on three main aspects: firstly, researchers have explored the relationship between population influx and infectious diseases. Population influx can lead to the spread of infectious diseases (1, 2). For example, when people move from one region to another, they may carry infectious disease pathogens such as HIV/AIDS, tuberculosis, hepatitis, malaria, and COVID-19, which can cause outbreaks of infectious diseases (3, 4). This movement can disrupt the ecological balance of diseases in new regions (5). When a large number of people migrate to an area with lower disease prevalence, increased contact with the native population allows new pathogens to spread locally, potentially causing an imbalance in the existing disease ecology, as the native population may not have developed immunity to these diseases (6, 7). Population influx also presents significant challenges to the prevention and control of infectious diseases (8). The characteristics of population influx, such as dispersed residence, complicate monitoring and prevention efforts (9). Additionally, population agglomeration can result in shortages of medical resources and increased pressure on prevention and control system, which further aggravates the difficulty of prevention and control of infectious diseases (10, 11).

Secondly, some researchers have studied the relationship between population influx and air pollution (12, 13). As population increases, energy consumption in newly populated regions also rises (14, 15). This heightened energy consumption leads to increased emissions of greenhouse gasses and other air pollutants, such as particulate matter and sulfur dioxide (16, 17). Population influx can alter land use patterns, disrupting established natural ecosystems, reducing urban green spaces, and decreasing vegetation’s capacity to absorb air pollutants (18, 19). Furthermore, increased demand for agricultural land due to emigration further exacerbates the impact of land use changes on air quality (20).

Thirdly, some researchers have studied the relationship between air pollution and infectious diseases (21, 22). Air pollution significantly impacts respiratory infectious diseases (23, 24). Particulate matter and harmful pollutants can irritate and damage respiratory mucosa, reduce the defensive function of the respiratory tract, and increase susceptibility to respiratory infections. Meanwhile, air pollution can affect the balance of intestinal microbial flora, potentially increasing susceptibility to intestinal infections (25). Furthermore, air pollutants can enhance the stability and transmission range of viruses, thus increasing their infection rate (26, 27). Individuals exposed to air pollution for extended periods may face a higher risk of contracting infectious diseases, resulting in increased mortality rate (28).

In conclusion, the existing studies have primarily focused on the bivariate relationships among population influx, air pollution, and infectious diseases. However, few studies have explored the interactions and logical relationships among all three variables. This paper aims to explore the mechanism through which population influx impacts infectious diseases, with air pollution as a mediating variable. We construct several econometric models to empirically test the health risks posed by population influx. The main contributions of this paper are as follows: Firstly, we analyze the direct and indirect effects of population influx on infectious diseases from an epidemiological perspective. Secondly, we examine regional heterogeneity to determine whether the risk of infectious diseases due to population influx varies across different regions. Thirdly, we analyze the influence of personal income, medical resources, and educational resources on the risk of infectious diseases associated with population influx from economic and social perspectives.

## 2 Theoretical analysis and research hypotheses

**Figure 1:**
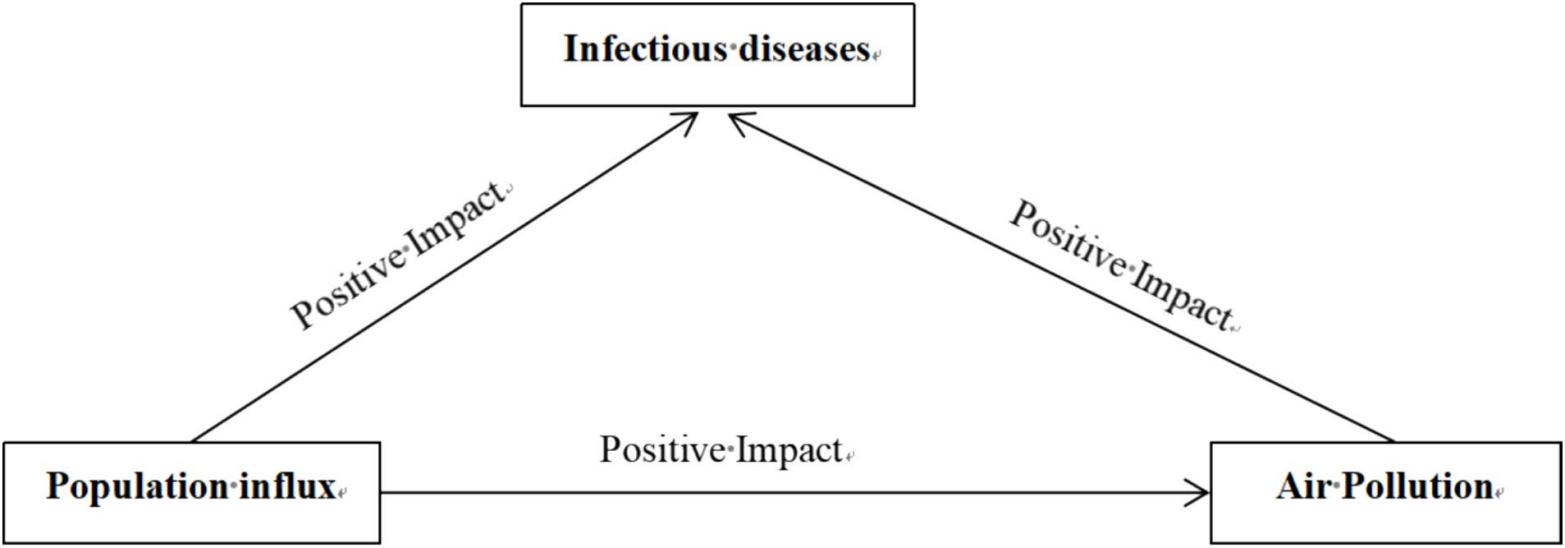
Direct and indirect effects of population influx on infectious diseases.

Population influx can serve as a vector for pathogen transmission, crossing natural geographical barriers or spreading via airborne particles in polluted air, as shown in Figure 1. Immigrants can introduce pathogens into new ecological or geographical locations. These pathogens can attach to the immigrants or their belongings and begin to spread in the new region (29). During the initial stages, immigrants may not exhibit any symptoms or may only present mild symptoms, making it difficult to identify them as potential disease transmitters (8, 30). Given their regular engagement in routine social activities and interactions with others, immigrants can act as carriers of pathogens, transmitting them to others. Pathogens have the ability to evolve in new environments, producing more adaptive mutant strains (31). These mutated strains may become more infectious by developing resistance to drugs or evading the host’s immune system (32). In addition to direct human-to-human transmission, pathogens can also be transmitted through interactions with the surrounding air, particularly through aerosols (33, 34). These pathogens can propagate, increasing the risk of airborne diseases (7). Long-term exposure to air pollutants may weaken the immune function, reduce the defense ability of mucosal barrier, and induce airway inflammation and oxidative stress, aggravating the severity of existing infection and promoting the spread and outbreak of infectious diseases (26, 35). Therefore, this paper proposes:

*Hypothesis 1*: Population influx can not only directly lead to the spread of infectious diseases, but may also indirectly contribute to their spread through air pollution.

**Figure 2:**
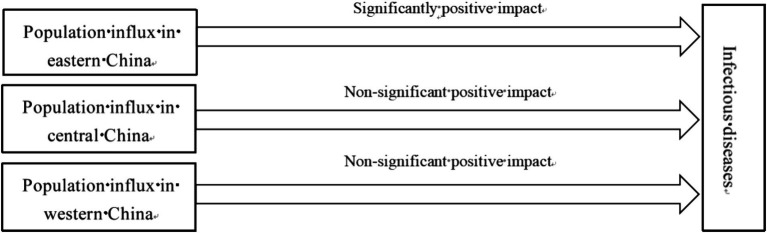
The risk of infectious diseases caused by population influx in eastern, central and western China.

There is regional heterogeneity in the risk of infectious diseases caused by population influx, as shown in Figure 2. Compared to the central and western regions of China, the eastern regions, as the country’s economic center, have higher levels of urbanization and faster economic development, which attracts a large number of migrants from the interior (36). The population agglomeration in the eastern regions not only increases the risk of infectious diseases, but also strains medical resources, leading to shortages. This may result in medical institutions being unable to prevent and control sudden outbreaks of infectious diseases effectively, thereby increasing the risk of disease transmission (37). Excessive urbanization due to population influx can exacerbate air pollution in the eastern regions, aggravating respiratory diseases such as bronchitis and asthma, which in turn increases the risk of spreading infectious respiratory diseases (38, 39). Poor air quality can also weaken residents’ immunity, making them more susceptible to various pathogens. Therefore, this paper proposes:

*Hypothesis 2*: Population influx has significantly promoted the spread of infectious diseases in the eastern regions of China, while the risk of infectious diseases caused by population influx is relatively low in the western regions.

**Figure 3:**
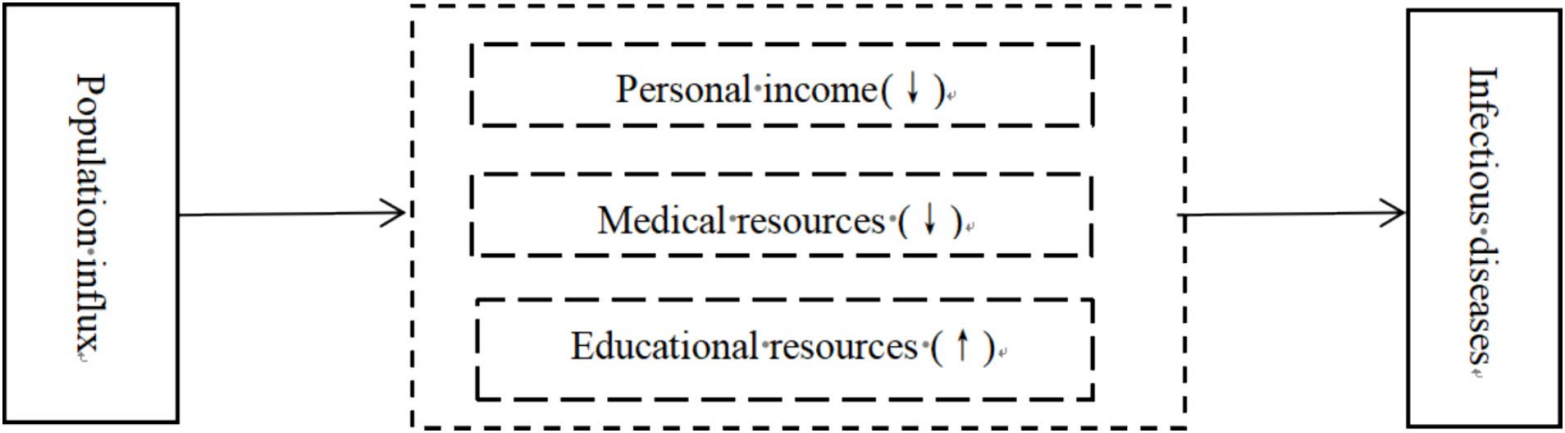
Impact of personal income, medical resources and educational resources on the risk of infectious diseases caused by population influx.

Personal income, medical resources, and educational resources exert varying degrees of impact on the risk of infectious diseases resulting from population influx, as shown in Figure 3. Residents in high-income regions usually have access to superior medical services, including more hospitals, clinics, skilled medical professionals, and advanced medical equipment. This enables prompt detection and treatment of infectious diseases, thereby mitigating virus transmission (40). Moreover, individual in these regions often benefit from better education, possess heightened health awareness, and are more adept at implementing preventive measures during infectious disease outbreaks (41). Therefore, this paper proposes:

*Hypothesis 3.1*: An increase in personal income can reduce the risk of infectious diseases caused by population influx.

Regions with abundant medical resources typically possess advanced medical equipment and highly skilled medical teams, facilitating early detection and diagnosis of diseases (42). When individuals migrate to these regions, despite potentially carrying pathogens, early diagnosis and treatment can effectively mitigate disease spread and minimize the impact on local residents’ health (43). During infectious diseases outbreaks, these regions can implement stringent measures to isolate patients, thereby preventing further epidemic spread. In addition, their robust treatment capabilities contribute to higher patient recovery rates and reduced mortality (44). Therefore, this paper proposes:

*Hypothesis 3.2*: The agglomeration of medical resources can reduce the risk of infectious diseases caused by population influx.

Abundant regional educational resources in a region can attract families to relocate, thereby increasing population density. This heightened density fosters more frequent interpersonal contact, potentially elevating the risk of infectious diseases (45). In densely populated regions, especially during periods of heightened infectious disease prevalence, students arriving from diverse geographic areas may introduce various pathogens, increasing the likelihood of new infectious strains emerging on campus. Therefore, this paper proposes:

*Hypothesis 3.3*: The concentration of educational resources increase the risk of infectious diseases caused by population influx.

## 3 Model and material

### 3.1 Model development

To assess the impact of population influx on infectious diseases, this paper constructs the following models:


(1) $$lnInfect_{it}=\alpha_{0}+\alpha_{1}PInflux_{it}+\alpha_{2}Controls+\lambda_{i}+\delta_{t}+\varepsilon_{it}$$

(2) $$\ln SO_{2it}=\beta_{0}+\beta_{1}PInflux_{it}+\beta_{2}Controls+\lambda_{i}+\delta_{t}+\varepsilon_{it}$$

(3) $$\begin{array}{l} lnInfect_{it}=\gamma_{0}+\gamma_{1}PInflux_{it}+\gamma_{2}\ln SO_{2it}+\\ \gamma_{3}Controls+\lambda_{i}+\delta_{t}+\varepsilon_{it} \end{array}$$

(4) $$\begin{array}{l} lnInfect_{it}=\delta_{0}+\delta_{1}PInflux_{it}\times I\left(q_{i,t}\leq T\right)+\delta_{2}PInflux_{it}\times \\ I\left(q_{i,t}>T\right)+\delta_{3}Controls+\delta \lambda_{i}+\delta_{t}+\varepsilon_{it} \end{array}$$

We construct a mediating model using Equations 1–3, corresponding, respectively, to Models 1–3 in Table 1, with Equation (1) serving as the baseline model employing two-way fixed effects. Equation (4) is the threshold model, and its regression results are presented as Models 11–13 in Table 2. In the above econometric models, i denotes the province, t indicates the year, $lnInfect_{it}$ represents infectious diseases as the explained variable, $PInflux_{it}$ stands for population influx as the explanatory variable, $\ln SO_{2it}$ acts as the mediating variable representing air pollution, T denotes the threshold value, $q_{i,t}$ signifies the threshold variable, Controls encompasses a set of control variables, $\lambda_{i}$ denotes province-fixed effect, $\delta_{t}$ represents time-fixed effect, and $\varepsilon_{it}$ is the random disturbance term.

**Table 1:** Regression results of the impact and mediating mechanism of population influx on infectious diseases.

Variables	Model 1	Model 2	Model 3
	lnInfect	lnSO_2_	lnInfect
	Coefficient(Se)	Coefficient(Se)	Coefficient(Se)
PInflux	0.418^**^(0.167)	0.608^*^(0.354)	0.365^**^(0.161)
lnSO_2_			0.088^***^(0.026)
Constant	3.859^***^(0.619)	−1.546(1.294)	3.985^***^(0.586)
Control variables	Yes	Yes	Yes
Province	Yes	Yes	Yes
Year	Yes	Yes	Yes
Observation	341	341	341

Standard errors are presented in parentheses, and the symbols “*,” “**,” and “***” are used to indicate the 10, 5, and 1% significance levels, respectively. The same labeling is employed in the subsequent sections of this paper.

**Table 2:** Regression results of threshold effect.

Variables	Model 4	Model 5	Model 6
	lnInfect	lnInfect	lnInfect
	Coefficient(Se)	Coefficient(Se)	Coefficient(Se)
PInflux (lnActpergdp ≤ 79992.422)	0.452^***^(0.166)		
PInflux (lnActpergdp > 79992.422)	0.327^*^(0.184)		
PInflux (Hospital ≤ 9,495)		1.707^***^(0.416)	
PInflux (Hospital > 9,495)		0.401^**^(0.160)	
PInflux (College ≤ 0.029)			0.388^**^(0.160)
PInflux (College > 0.029)			1.400^***^(0.334)
Constant	3.931^***^(0.599)	3.934^***^(0.585)	4.054^***^(0.587)
Control variables	Yes	Yes	Yes
Province	Yes	Yes	Yes
Year	Yes	Yes	Yes
Observation	341	341	341

### 3.2 Variables setting and data sources

#### 3.2.1 Explained variable

Infectious diseases (*lnInfect*). We select the incidence rate of legally reported Class A and Class B infectious diseases as proxy indicators. This rate denotes the number of Class A and Class B infectious diseases reported per 100,000 people within a specific region and year. Mathematically, it is calculated as the number of legally reported infectious diseases in Class A and Class B divided by the population, multiplied by 100,000. That is, the number of legally reported infectious diseases = number of legally reported infectious diseases in Class A and Class B/population × 100,000. Data for this study are sourced from the China Healthcare Statistics Yearbook, covering the period from 2010 to 2020 and comprising provincial panel data. All data are obtained directly from the official yearbooks published by the National Bureau of Statistics, thereby ensuring the data’s authenticity and accuracy. Furthermore, the interpolation method is employed to fill in the missing data.

#### 3.2.2 Explanatory variable

Population influx (*PInflux*). We use the sum of registered residence immigration and non-registered residence immigration as the proxy indicator, defined as population immigration = registered residence immigration + non-registered residence immigration. Registered residence immigration volume is calculated using the formula: registered residence immigration volume = registered residence population in the current year – registered residence population at the end of last year – average registered residence population × natural population growth rate. Non-registered residence immigration is calculated as: non-registered residence immigration = number of permanent residents in the current year – number of registered residence residents in the current year – number of permanent residents in the previous year – number of registered residence residents in the previous year. The above data is sourced from the China Statistical Yearbook, covering the period from 2010 to 2020 and comprising provincial panel data. All data are directly from the official yearbooks published by the National Health and Wellness Commission of China, ensuring their authenticity and accuracy. Missing data are addressed using interpolation methods.

#### 3.2.3 Mediating variable

Air pollution (*lnSO_2_*). We use the concentration of SO_2_ as a proxy indicator of air pollution. Since 2000, the Chinese government has designated 175 cities as air pollution control zones to regulate sulfur dioxide emissions and prevent acid rain formation. This initiative aims to enhance air quality, safeguard the ecological environment, and promote human health. Additionally, the concentration of sulfur dioxide can be easily monitored through monitoring stations. The data mentioned above are sourced from the China Environmental Yearbook, covering the period from 2010 to 2020 and comprising provincial panel data. All data are sourced directly from the official yearbooks published by the PRC Ministry of Ecology and Environment (MEE), ensuring their authenticity and accuracy. Missing data are addressed using interpolation methods.

#### 3.2.4 Threshold variables

Personal income (*lnActpergdp*). Real GDP *per capita* is selected as a proxy for personal income because it serves as a crucial indicator of a region’s economic status, reflecting the living standards and incomes of its residents. For this study, we use 2010 as the base year and 2020 as the reporting year, adjusting nominal GDP *per capita* with a deflator to derive real GDP *per capita*. Data on personal income are sourced from the China Statistical Yearbook. Medical Resources (*Hospital*). We select healthcare institutions as medical resources because they play a vital role in diagnosing diseases and effectively managing the risk of infectious diseases. Data on medical resources are sourced from the China Healthcare Statistics Yearbook. Educational resources (*College*). The ratio of regional universities to the total number of universities nationwide is selected as a proxy indicator for educational resources. This metric not only reflects public health awareness, but also indicates the regional share in the allocation of higher education resources across the country. Data on educational resources are sourced from the China Education Statistical Yearbook. The dataset spans from 2010 to 2020 and includes provincial panel data, ensuring the authenticity and accuracy of the data sourced directly from official yearbooks published by the respective Chinese government agencies. Interpolation methods are employed to address any missing data.

#### 3.2.5 Control variables

This paper incorporates a comprehensive set of control variables for infectious diseases based on previous studies. The main variables are as follows: (1) Commercial health insurance (*Insur*). We select the ratio of commercial health insurance premium income to total population as a proxy indicator for commercial health insurance. This metric compensates patients for income loss and medical expenses due to infectious diseases, providing extensive health protection to insured individuals. (2) Urbanization (*Urban*). The ratio of urban population to total population serves as a proxy indicator for urbanization. This is because urbanization often correlates with population density and air quality, which can accelerate the transmission and outbreak of infectious diseases. (3) *Per capita* Park green region (*Pergygreen*). We choose the ratio of park green space region to total population as the proxy indicator for *per capita* park green space. This is because it can promote outdoor activities, enhances immunity, improves public health, and mitigates the risk of infectious diseases. (4) *Per capita* healthcare consumption expenditure of residents (*Heaexp*). The proportion of residents’ healthcare consumption expenditure to the total population is selected as the proxy indicator for *per capita* healthcare consumption expenditure. This is because the greater increased investment in health and medical care improves individual immunity, thereby reducing the risk of infectious diseases. (5) Internet penetration rate (*Internet*). The ratio of the number of Internet users to the total population is used as a proxy indicator for Internet penetration. This is because a higher Internet penetration rate facilitates access to health knowledge and information, enhancing public awareness and preparedness against infectious disease outbreaks. (6) Public Toilet Penetration Rate (*Toilet*). The number of public toilets per 10,000 people serves as a proxy indicator for the public toilet penetration rate. This is because hygiene conditions in public toilets impact pathogen growth and transmission, increasing the risk of infectious diseases. Data for these variables are sourced from the China Statistical Yearbook. The data set spans the period from 2010 to 2020 and comprises provincial panel data, spanning from 2010 to 2020 and encompassing provincial panel data. All data are obtained directly from official yearbooks published by the National Bureau of Statistics, thereby ensuring their accuracy and authenticity. Interpolation methods are applied to address any missing data points.

## 4 Results

### 4.1 Benchmark regression and mediating mechanism test

According to Equations 1–3, we examine the impact of population influx on the incidence rate of infectious diseases, with detailed results presented in Table 1. It can be seen from Model 1 that the coefficient of PInflux is 0.418, passing the 5% significance level test, indicating that for each additional unit of migrant population in a destination, the incidence rate of infectious diseases increases by 0.418 units. This finding suggests a direct association between population influx and the occurrence of infectious diseases. Model 2 shows that the coefficient of PInflux is 0.608, passing the 10% significance level test, signifying that each additional unit of migrant population contributes to a 0.608 increase in air pollution. This indicates that population influx exacerbates air pollution levels. In Model 3, the coefficient for PInflux (0.365) and lnSO_2_ (0.088) also pass the 5% significance level test. This result demonstrates that population influx may exacerbate air pollution, thereby potentially increasing the incidence of infectious diseases. Notably, air pollution acts as a partial mediator, accounting for 12.8% of the total effect. Thus, Hypothesis 1 of this paper is supported by empirical evidence.

### 4.2 Robustness test

To ensure the reliability of the estimation results, this paper uses the systematic GMM estimation method and conducts robustness tests by substituting key variables, as shown in Table 3.

**Table 3:** Regression results of robustness analysis.

Variables	Model 7	Model 8	Model 9	Model 10
	lnInfect	lnInfect	lnInfect	lnInfect
	Coefficient(Se)	Coefficient(Se)	Coefficient(Se)	Coefficient(Se)
L. lnInfect	1.008^***^(0.055)			
PInflux	0.234^**^(0.092)	0.074^*^(0.040)	0.417^**^(0.163)	0.371^**^(0.161)
PerpInflux				
Constant	0.838(0.874)	−0.314^**^(0.145)	4.004^***^(0.691)	
Peredu			−0.021(0.047)	−0.020(0.046)
Waterzb				−1.236^***^(0.382)
Sargan *P*-value	0.219			
AR(1) *P*-value	0.002			
AR(2) *P*-value	0.364			
Control variables	Yes	Yes	Yes	Yes
Province	Yes	Yes	Yes	Yes
Year	Yes	Yes	Yes	Yes
Observation	341	341	341	341

#### 4.2.1 System GMM

To address endogeneity concerns, system GMM is used for regression estimation. The results are shown in Model 7. The Arellano Bond test indicates a significant *p*-value for AR (1) and a non-significant *p*-value for AR (2), suggesting the absence of second-order autocorrelation in the random disturbance term. The Sargan test yields a *p*-value greater than 0.1, confirming the validity of the selected instrumental variables. The coefficient of PInflux is 0.234, passing the 5% significance level test. This finding aligns with the conclusions drawn from the baseline regression estimates.

#### 4.2.2 Replacement of key variables

Given the ineffectiveness of antiviral drugs against certain infectious diseases and the potential for pathogens to produce potent toxins—such as pneumococcus causing severe lung infections, botulism blocking nerve signaling, HIV compromising the immune system, and COVID-19 being highly contagious with severe complications—the emergence of mutant strains complicates treatment, elevating mortality risks. Therefore, we select the mortality rate of infectious diseases as the explanatory variable. Model 8 shows a coefficient of 0.074 for PInflux, significant at the 10% level. This result underscores that population influx increases the mortality rate of infectious diseases, which is consistent with the results of the baseline regression.

#### 4.2.3 Gradual addition of control variables

To further ensure the robustness of our regression results against control variables, we introduced government education investment to the original control variables and obtain the regression results of Model 9. Here, the coefficient of PInflux is 0.417, which has passed the 5% significance level test. Subsequently, we incorporated the prevalence of tap water into Model 9 and obtain the regression results of Model 10, where the coefficient of PInflux is 0.371, also significant at the 5% level. These findings consistently indicate that population influx positively influences infectious diseases.

### 4.3 Heterogeneity analysis

From the regional heterogeneity perspective, we analyze the impact of population influx on infectious diseases in the eastern, central and western regions of China. Table 4 shows the regression results: Model 11 for the eastern regions, Model 12 for the central regions, and Model 13 for the western regions. The coefficient of PInflux in Model 11 is 0.535, which has passed the 5% significance level test. It means that each additional unit of migrant population increases the incidence rate of infectious diseases by 0.535 units in the eastern regions. This suggests that population influx in eastern China significantly raises the risk of infectious diseases. In contrast, the coefficients of PInflux in Model 12 and Model 13 are 0.283 and 0.711, respectively, but neither passes the significance level test, implying a potential but statistically insignificant risk of infectious diseases due to population influx in central and western China. Therefore, Hypothesis 2 of this paper is validated.

**Table 4:** Regression results of regional heterogeneity.

Variables	Model 11	Model 12	Model 13
	lnInfect	lnInfect	lnInfect
	Coefficient(Se)	Coefficient(Se)	Coefficient(Se)
PInflux	0.535^**^(0.220)	0.283(0.569)	0.711(0.456)
Constant	2.781^**^(1.300)	6.006^*^(3.223)	4.514^***^(1.071)
Control variables	Yes	Yes	Yes
Province	Yes	Yes	Yes
Year	Yes	Yes	Yes
Observation	143	66	132

### 4.4 Threshold effect

According to Equation (4), this paper further uses a threshold effect model to test the impact of personal income, medical resources, and educational resources on the risk of infectious diseases due to population influx. The estimation results are presented in Table 2. Firstly, using personal income as the threshold variable, Model 4 shows that when lnActbergdp ≤ 79992.422, the coefficient of PInflux is 0.452. When lnActpergdp > 79992.422, the coefficient of PInflux decreases to 0.327. Both coefficients have passed the 10% significance level test. This indicates that an increase in personal income can effectively reduce the risk of infectious diseases caused by population influx. Therefore, Hypothesis 3.1 of this paper is validated.

Secondly, using medical resources as a threshold variable, Model 5 reveals when Hospital ≤ 9,495, the coefficient of PInflux is 1.707. When Hospital > 9,495, the coefficient value of PInflux decreases to 0.401. Both coefficients have passed the 5% significance level test. This suggests that abundant medical resources can effectively reduce the risk of infectious diseases caused by population influx. Thus, Hypothesis 3.2 of this paper is validated.

Thirdly, using educational resources as a threshold variable, Model 6 shows that when College ≤ 0.029, the coefficient of PInflux is 0.388. When College > 0.029, the coefficient of PInflux increases to is 1.400. Both coefficients have passed the 5% significance level test. It demonstrates that abundant regional educational resources can effectively reduce the risk of infectious diseases caused by population influx. Hence, Hypothesis 3.3 of this paper is validated.

## 5 Discussion

Through the empirical tests conducted, we have identified several key findings: Firstly, population influx can not only directly lead to the spread of infectious diseases, but may also indirectly trigger epidemics through polluted air. However, direct transmission remains the primary means of disease spread. Notably, if the migrant population carries more virulent pathogens into new regions, the fatality rate of infectious diseases may rise significantly. The main reason for this is that the rapid spread of a new pathogen in a previously unexposed population can lead to an outbreak of infectious disease. Direct transmission routes are particularly prone to causing outbreaks and epidemics due to their rapid and widespread transmission characteristics. Pathogens may evolve more virulent mutant strains to evade the host’s immune system, thereby increasing the lethality of infectious diseases.

Secondly, the risk of infectious diseases caused by population influx in western China is not significant. This is primarily due to the low population density and sparse distribution in these regions, which reduces the frequency of contact between individuals and, consequently, the risk of disease transmission. Unlike the eastern regions of China, the western regions have less developed transportation infrastructure and a slower rate of population influx, further diminishing the potential for disease spread. Additionally, the air quality in central and western regions of China is superior to that in the east, weakening the indirect risk effect caused by population influx and thus lowering the incidence of infectious diseases.

Thirdly, personal income and medical resources can reduce the risk of infectious diseases caused by population influx. Notably, medical resources have a greater impact on reducing this risk. While personal income can lower the risk by improving living conditions and raising health awareness, its effect is relatively indirect and limited. In contrast, regions with abundant medical resources can promptly address disease outbreaks or public health emergencies, providing necessary medical services to both migrants and local residents, thereby preventing the further spread of diseases.

Fourthly, an excessive concentration of educational resources increases the risk of infectious diseases caused by population influx. Although enhancing educational resources improves residents’ health awareness and helps reduce the risk of infectious diseases, it also leads to a higher population density. This increased concentration of people subsequently elevates the risk of infectious disease transmission.

This paper shares several similarities with existing studies. Both highlight that population influx increases the risk of infectious diseases in the destination area due to factors such as increased population density, high social contact, and diverse immune statuses among the local population. Additionally, both this paper and existing research recognize that air pollution can contribute to the spread of infectious diseases. Airborne microorganisms, including bacteria, viruses, molds, and dust mites, as well as pathogens like COVID-19, can enter the human respiratory tract via suspended airborne particles, leading to respiratory diseases. These microorganisms can parasitize and multiply on the mucous membranes of the respiratory tract, causing illnesses such as colds, influenza, and pneumonia (46–48). However, this paper diverges from existing studies in a key aspect: while previous research suggests that abundant educational resources reduce the risk of infectious diseases due to increased health awareness, this paper finds the opposite. It argues that abundant educational resources can actually increase the risk of infectious diseases associated with in-migration. This is because better educational resources attract more families and students to the area, thereby increasing population density and the associated risk of disease transmission.

This paper focuses on macro-level data from 31 provinces in China, revealing the impact and mechanisms of population influx on the risk of infectious diseases at the regional level. However, it inevitably ignores micro-level heterogeneity. Specifically, it does not address the risk of infectious diseases brought by population influx to different demographic groups. Future research should concentrate on empirical analysis at the micro level, distinguishing among different population groups to analyze the risk of infectious diseases affected by population influx at the individual level.

## 6 Conclusions and policy recommendations

### 6.1 Conclusion

Population influx is a phenomenon that not only relocates people and alters their living environments but also has a far-reaching impact on the prevalence and spread of infectious diseases (49, 50). In the context of globalization and urbanization, this paper conducts a thorough analysis of the relationships among population influx, air pollution, and infectious diseases. The aim is to provide insights for the formulation of effective public health policies and social risk management measures (51).

The following three research conclusions are drawn from this paper. Firstly, population influx can directly cause infectious diseases and also indirectly contribute to their spread through polluted air, although direct contact between migrants and local populations remains the primary transmission route. In addition, the incidence of infectious diseases caused by population influx is greater than the mortality rate of infectious diseases. Secondly, population influx in eastern regions of China has elevated the incidence of infectious diseases beyond the national average. In contrast, migration from central and western regions has posed only a potential risk of infectious diseases. Thirdly, personal income and medical resources can reduce the risk of infectious diseases caused by population immigration. However, medical resources are more effective in reducing this risk compared to personal income. Fourthly, contrary to expectations, abundant educational resources do not decrease the risk of infectious diseases but rather exacerbates the risk of infectious diseases caused by immigration.

### 6.2 Policy recommendations

Accordingly, we put forward the following three policy recommendations. Firstly, the government should formulate a more scientific and rational population relocation policy that balances population influx with urban carrying capacities, thereby preventing over-concentration of migration in China’s eastern coastal regions. Enhanced management and services for migrants are essential, including initiatives to improve their health awareness and self-protection skills.

Secondly, the government should prioritize improving air quality in eastern China by intensifying efforts to combat industrial pollution and reduce air pollution levels. In addition, the government should expand the healthcare infrastructure and improve medical services in this region. Establishing a robust infectious disease prevention and control system is crucial to ensure prompt diagnosis, isolation, and treatment of infectious disease cases. Moreover, the government should strengthen infectious disease prevention and health education initiatives specifically tailored for residents in western China. Enhancing public health awareness and knowledge about infectious disease prevention among western residents is essential.

Thirdly, the government should raise the income levels of the impoverished by generating employment opportunities, providing training programs, and establishing robust social security and welfare systems. These measures can help alleviate the healthcare burden on individuals. Furthermore, the government should increase investments in medical resources in regions where the population is concentrated, strengthen public health services and infrastructure, and improve mechanisms for infectious disease prevention. This strategy aims to ensure widespread access to timely medical care and mitigate the incidence of infectious diseases.

Fourthly, the government should reassess its educational resource allocation strategy to ensure both adequacy and equitable distribution across counties. This will mitigate the over-concentration of educational resources in certain regions and reduce the potential for large-scale population influx triggered by unequal access to education. In regions with plentiful educational resources, integrating public health education programs into school curricula can enhance awareness among students and parents regarding infectious disease prevention. Schools are also encouraged to collaborate with local health authorities in health promotion activities.

## Data availability statement

The original contributions presented in the study are included in the article/supplementary material, further inquiries can be directed to the corresponding author.

## Author contributions

HF: Data curation, Methodology, Resources, Writing – original draft. CZ: Writing – review & editing, Data curation, Formal analysis, Funding acquisition.
