# Corrigendum: The impact of population influx on infectious diseases – from the mediating effect of polluted air transmission

**DOI:** 10.3389/fpubh.2024.1480516

**Published:** 2024-09-03

**Authors:** Haifeng Fu, Chaoping Zhu

**Affiliations:** ^1^School of Accounting and Finance, Jiangxi College of Foreign Studies, Nanchang, Jiangxi, China; ^2^School of Transportation Management, Jiangxi Vocational and Technical College of Communications, Nanchang, Jiangxi, China; ^3^School of Software, Jiangxi Normal University, Nanchang, Jiangxi, China

**Keywords:** population influx, air pollution, infectious diseases, personal income, medical resources, educational resources

In the published article, there was an error regarding the affiliation(s) for (Haifeng Fu). As well as having affiliation(s) (*School of Transportation Management, Jiangxi Vocational and Technical College of Communications, Nanchang, Jiangxi, China*), they should also have (^1^ *School of Accounting and Finance, Jiangxi College of Foreign Studies, Nanchang, Jiangxi, China*).

The authors apologize for this error and state that this does not change the scientific conclusions of the article in any way. The original article has been updated.

